# Supplementary material for: Changes in Dietary Nutrient Intake and Estimated Glomerular Filtration Rate over a 5-Year Period in Renal Transplant Recipients
Source: Nutrients. 2023 Dec 31;16(1):148. doi: 10.3390/nu16010148 (PMC10780404; doi:10.3390/nu16010148)
Supplement: Supplementary file 1 [file nutrients-16-00148-s001.zip › nutrients-2774755-supplementary.pdf]

Supplementary Table S1. Demographic, anthropometric, clinical, and nutritional data of 227 RTRs [1,2]

| | ALL | T1 stage | T2 stage | | | ALL | T1 stage | T2 stage | |
|---|---|---|---|---|---|---|---|---|---|
| Numbers | 227 | 85 | 142 | *P* value | | 227 | 85 | 142 | *P* value |
| **Demographics** | | | | | **Dietary intake** | | | | |
| Male/female | 127/100 | 45/40 | 81/61 | | Energy, kcal/day | 1793.61 ± 457.02 | 1872.58 ± 377.8 | 1746.34 ± 493.70 | 0.031 |
| Age, year | 49.97 ± 12.30 | 49.72 ± 12.60 | 50.12 ± 12.16 | 0.812 | Carbohydrate, g/day | 191.46 ± 56.22 | 207.22 ± 47.34 | 182.02 ± 59.09 | 0.001 |
| Renal transplant time, year | 9.22 ± 7.91 | 8.83 ± 5.97 | 9.45 ± 8.88 | 0.532 | Carbohydrate, % energy | 42.82 ± 7.82 | 44.53 ± 6.46 | 41.79 ± 8.38 | 0.006 |
| Tacrolimus/cyclosporine used | 164/41 | 69/16 | 95/25[$] | | Protein, g/day | 68.88 ± 18.07 | 67.39 ± 14.06 | 69.77 ± 20.09 | 0.295 |
| Deceased/living donors | 156/71 | 69/16 | 87/55 | | Protein, % energy | 15.55 ± 2.68 | 14.46 ± 1.76 | 16.21 ± 2.91 | <.0001 |
| **Anthropometry** | | | | | Fat, g/day | 83.63 ± 26.99 | 84.89 ± 22.41 | 82.88 ± 29.44 | 0.562 |
| Height, cm | 162.26 ± 8.92 | 161.39 ± 8.61 | 162.79 ± 9.09 | 0.254 | Fat, % energy | 41.72 ± 6.71 | 40.55 ± 5.71 | 42.41 ± 7.17 | 0.032 |
| Body weight, kg | 63.26 ± 13.91 | 62.88 ± 13.26 | 63.49 ± 14.33 | 0.750 | SFA, g/day | 21.30 ± 8.56 | 19.47 ± 7.34 | 22.40 ± 9.06 | 0.009 |
| Body mass index, kg/m$^2$ | 23.87 ± 4.04 | 24.00 ± 3.83 | 23.79 ± 4.18 | 0.707 | SFA, % energy | 10.59 ± 2.73 | 9.30 ± 2.72 | 11.36 ± 2.44 | <.0001 |
| **Laboratory** | | | | | MUFA, g/day | 30.11 ± 12.10 | 27.07 ± 9.21 | 31.93 ± 13.23 | 0.001 |
| Albumin, g/dL | 4.27 ± 0.31 | 4.34 ± 0.30 | 4.23 ± 0.31 | 0.017 | MUFA, % energy | 15.12 ± 4.59 | 13.02 ± 3.58 | 16.38 ± 4.68 | <.0001 |
| Blood urea nitrogen, mg/dL | 25.56 ± 11.75 | 24.05 ± 11.59 | 26.52 ± 11.79 | 0.129 | PUFA, g/day | 27.27 ± 12.58 | 26.98 ± 11.51 | 27.44 ± 13.22 | 0.792 |
| Creatinine, mg/dL | 1.47 ± 0.90 | 1.43 ± 0.76 | 1.49 ± 0.98 | 0.587 | PUFA, % energy | 13.65 ± 5.19 | 12.93 ± 4.79 | 14.09 ± 5.39 | 0.102 |
| Total cholesterol, mg/dL | 206.41 ± 49.05 | 208.20 ± 45.34 | 205.30 ± 51.35 | 0.669 | Cholesterol, mg/day | 282.13 ± 136.58 | 247.58 ± 127.85 | 302.80 ± 137.88 | 0.003 |
| Triglycerides, mg/dL | 152.93 ± 112.04 | 157.92 ± 122.19 | 149.77 ± 105.46 | 0.601 | Fiber, g/day | 12.95 ± 5.73 | 13.16 ± 5.37 | 12.82 ± 5.95 | 0.667 |
| HDL-C, mg/dL | 55.78 ± 17.91 | 52.25 ± 17.79 | 58.08 ± 17.69 | 0.019 | Sodium, mg | 1180.05 ± 1417.23 | 1029.83 ± 687.99 | 1269.97 ± 1707.52 | 0.139 |
| HbA1c, % | 6.05 ± 1.01 | 6.06 ± 1.01 | 6.04 ± 1.01 | 0.863 | Calcium, mg/day | 434.99 ± 260.63 | 347.60 ± 163.18 | 487.30 ± 292.67 | <.0001 |
| Insulin, U/mL | 12.57 ± 41.01 | 8.56 ± 13.04 | 15.43 ± 52.47 | 0.173 | Magnesium, mg/day | 250.58 ± 377.62 | 296.79 ± 600.54 | 222.91 ± 107.96 | 0.264 |

| | | | | | | | | | |
|---|---|---|---|---|---|---|---|---|---|
| Uric acid, mg/dL | 5.92 ± 1.35 | 6.20 ± 1.33 | 5.74 ± 1.34 | 0.013 | Phosphurous, mg/day | 829.34 ± 286.24 | 726.98 ± 227.99 | 890.61 ± 300.44 | <.0001 |
| hs-CRP, mg/dL | 4.94 ± 12.12 | 5.16 ± 12.20 | 4.79 ± 12.11 | 0.830 | Potassium, mg/day | 1998.93 ± 741.40 | 1791.88 ± 634.45 | 2122.86 ± 774.54 | 0.001 |
| **Six primary food categories** | | | | | Iron, mg/day | 9.56 ± 5.15 | 8.86 ± 2.57 | 9.98 ± 6.17 | 0.058 |
| Whole grains and cereals, servings/day | 9.52 ± 3.18 | 10.56 ± 2.69 | 8.89 ± 3.30 | <.0001 | Zinc, mg/day | 8.74 ± 2.89 | 8.66 ± 2.59 | 8.79 ± 3.07 | 0.748 |
| Soybeans, fish, eggs, and meat, servings/day | 6.28 ± 2.23 | 5.78 ± 1.62 | 6.58 ± 2.48 | 0.004 | Vitamin B1, mg/day | 1.00 ± 0.45 | 1.04 ± 0.35 | 0.98 ± 0.51 | 0.309 |
| Dairy products, servings/day | 0.30 ± 0.54 | 0.19 ± 0.34 | 0.36 ± 0.63 | 0.010 | Vitamin B2, mg/day | 0.97 ± 0.39 | 0.87 ± 0.27 | 1.02 ± 0.43 | 0.001 |
| Vegetables, servings/day | 2.54 ± 1.47 | 2.50 ± 1.05 | 2.57 ± 1.68 | 0.683 | Niacin, mg/day | 14.04 ± 5.53 | 12.16 ± 4.00 | 15.17 ± 6.00 | <.0001 |
| Fruits, servings/day | 1.07 ± 1.16 | 1.21 ± 1.02 | 0.99 ± 1.24 | 0.143 | Vitamin B6, mg/day | 1.47 ± 0.57 | 1.26 ± 0.37 | 1.59 ± 0.63 | <.0001 |
| Oils, fats, nuts and seeds, servings/day | 9.89 ± 3.72 | 10.01 ± 3.18 | 9.82 ± 4.02 | 0.689 | Vitamin B12, ug/day | 3.17 ± 2.66 | 2.94 ± 1.88 | 3.30 ± 3.03 | 0.274 |
| **Others** | | | | | Folic acid, ug/day | 248.16 ± 109.76 | 207.81 ± 101.34 | 272.31 ± 107.81 | <.0001 |
| eGFR, ml/min/1.73 $m^2$ | 55.70 ± 21.77 | 54.71 ± 21.48 | 56.29 ± 22.00 | 0.598 | Vitamin C, mg/day | 119.75 ± 92.35 | 101.79 ± 61.89 | 130.50 ± 105.25 | 0.010 |
| SBP, mmHg | 136.50 ± 17.99 | 133.37 ± 15.98 | 138.45 ± 18.92 | 0.033 | Vitamin A, ug RE/day | 812.07 ± 744.74 | 787.49 ± 379.67 | 826.79 ± 895.85 | 0.064 |
| DBP, mmHg | 80.14 ± 12.91 | 77.90 ± 11.89 | 81.53 ± 13.36 | 0.042 | Vitamin E, mg α-TE/day | 12.55 ± 13.76 | 9.92 ± 3.83 | 14.12 ± 16.97 | 0.005 |
| HOMA-IR | 2.98 ± 11.03 | 2.35 ± 4.96 | 3.44 ± 13.93 | 0.449 | | | | | |

Abbreviations: RTRs, renal transplant recipients; HDL-C, high-density lipoprotein cholesterol; HbAlC, glycated hemoglobin A1c; hs-CRP, high-sensitivity C-reactive protein; eGFR, estimated glomerular filtration rate; SBP, systolic blood pressure; DBP, diastolic blood pressure; HOMA-IR, homeostasis model assessment–estimated insulin resistance; SFA, saturated fatty acid; MUFA, monounsaturated fatty acid; PUFA, polyunsaturated fatty acid; RE, retinol equivalent; TE, tocopherol equivalent.

[1] This two-stage cross-sectional study was conducted from September 2016 to June 2018, referred to as the T1 stage, followed by the T2 stage, spanning from July 2022 to August 2023. Data are presented as the mean ± standard deviation or number, as appropriate.

[2] Statistical analyses were conducted using Student's t test or Wilcoxon rank sum test, as appropriate.

[$]No records of 22 patients.
